# Detection of Functional Modes in Protein Dynamics

**DOI:** 10.1371/journal.pcbi.1000480

**Published:** 2009-08-28

**Authors:** Jochen S. Hub, Bert L. de Groot

**Affiliations:** Computational Biomolecular Dynamics Group, Max-Planck-Institute for Biophysical Chemistry, Göttingen, Germany; National Cancer Institute, United States of America and Tel Aviv University, Israel

## Abstract

Proteins frequently accomplish their biological function by collective atomic motions. Yet the identification of collective motions related to a specific protein function from, e.g., a molecular dynamics trajectory is often non-trivial. Here, we propose a novel technique termed “functional mode analysis” that aims to detect the collective motion that is directly related to a particular protein function. Based on an ensemble of structures, together with an arbitrary “functional quantity” that quantifies the functional state of the protein, the technique detects the collective motion that is maximally correlated to the functional quantity. The functional quantity could, e.g., correspond to a geometric, electrostatic, or chemical observable, or any other variable that is relevant to the function of the protein. In addition, the motion that displays the largest likelihood to induce a substantial change in the functional quantity is estimated from the given protein ensemble. Two different correlation measures are applied: first, the Pearson correlation coefficient that measures linear correlation only; and second, the mutual information that can assess any kind of interdependence. Detecting the maximally correlated motion allows one to derive a model for the functional state in terms of a single collective coordinate. The new approach is illustrated using a number of biomolecules, including a polyalanine-helix, T4 lysozyme, Trp-cage, and leucine-binding protein.

## Author Summary

Proteins are flexible nanomachines that frequently accomplish their biological function by collective atomic motions. Such motions may be characterized by hinge, shear, or rotational motions of entire protein domains, loop movements, or subtle rearrangements of amino acid side chains. In many cases it is far from obvious how collective motions are related to a particular biological task. Therefore, we propose a novel technique termed “functional mode analysis” that, based on an ensemble of structures, aims to detect a collective motion that is directly related to a particular protein function. From the given set of protein structures, together with a “functional quantity”, the technique seeks the collective motion that is maximally correlated to the functional quantity. The chosen functional quantity can be quite general; typical examples could include the openness of a channel, active site geometry, or cleft solvent accessibility. Because the proposed framework is highly general, we expect the approach to be useful to a wide range of applications. To illustrate the new technique, we apply functional mode analysis to molecular dynamics trajectories of a polyalanine-helix, bacteriophage T4 lysozyme, Trp-cage, and Leucine-binding protein.

## Introduction

Collective motions are essential for biological functions in proteins [1]. They are involved in numerous biological processes including enzyme catalysis, channel gating, allosteric interactions, signal transduction, and recognition dynamics. The observed motions are as diverse as hinge, shear, or rotational motions of entire subunits, opening motions of molecular lids, loop motions, partial unfolding, or subtle rearrangements of amino acid side chains [2]. Understanding the functional mechanisms of such proteins requires both to identify the protein's collective motions and to relate the observed motions to the protein's biological function.

Diverse experimental methods have been applied to elucidate collective motions including nuclear magnetic resonance (NMR) [3],[4], X-ray crystallography [5], as well as single-molecule fluorescence [6] or electron-transfer measurements [7]. Complementary to experiments, molecular dynamics (MD) simulations are a widely used techniques to investigate collective motions in proteins [8]. A state-of-the-art approach to elucidate collective motions from the protein dynamics is principal component analysis (PCA) [9]–[12]. PCA is commonly used to extract the collective motions with the largest contribution to the variance of the atomic fluctuations. Alternatively, normal mode analysis (NMA) has been extensively used to identify low-frequency collective modes [13]–[16]. Such modes are expected to correspond to large atomic displacements and are therefore assumed to be important to protein function. In addition, elastic network models are an established approach to assess motions intrinsic to the protein structure [17].

Established methods such as PCA and NMA elucidate large-scale and low-frequency modes, respectively, but do not necessarily yield collective motions directly related to protein function. Here, we propose a novel analysis technique termed ‘functional mode analysis’ (FMA) that aims to elucidate collective motions directly related to a specific protein function. As input, the technique requires a set of protein structures, together with a ‘functional quantity’ that can be expressed as a single number for each input structure. The structures typically derive from an MD simulation, but a (large) set of X-ray or NMR structures is equally well suited. The chosen ‘functional quantity’ can be quite general and could correspond to some geometric, electrostatic, or chemical observable, or any other variable that might be relevant to the function of the protein. Typical examples for the functional quantity could include the openness of a channel, active site geometry, or cleft solvent accessibility. Given that input, the technique seeks the collective protein motion that is maximally related to the functional quantity. In other words, the technique aims to explain variations in the functional quantity in terms of collective motions.

When relating a functional quantity (from now on termed 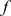) to collective motions, two quite different motions might be of interest. First, the motion that displays the largest correlation to 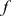. This motion is unaffected by the energy landscape of the protein, and it will be referred to as ‘maximally correlated motion’ (MCM). It is particularly interesting for quantities 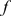 of which the dependence on the protein structure is complex and therefore unclear. An example for such a complex quantity would be the R-value in X-ray refinement. Second, the physical motion that actually accomplishes substantial deviations in 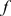, in accordance with the protein's energy landscape, is frequently of interest. Because many different motions might affect 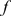, we use the input structure ensemble to estimate the most probable collective motion that accomplishes a substantial change in 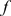. That motion will be referred to as ensemble-weighted MCM (ewMCM). Depending on the question addressed, the MCM or the ewMCM (or both) can provide insight into the relation between function and motion. Therefore, both motions are considered by the proposed framework.

This paper is organized as follows. First, we describe the analysis technique. Subsequently, four examples for FMA are presented, applying the approach to a polyalanine helix, T4 lysozyme, Trp-cage, and leucine-binding protein. The examples illustrate the use of FMA in detecting functionally relevant collective motions.

## Methods

### Theory and Concepts

Let us consider the simulation trajectory 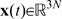 of the protein atoms or of a subset of the protein atoms such as the backbone or the heavy atoms. 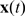 denotes the 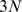 cartesian coordinates of 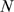 atoms. The coordinates are known at 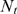 times, i.e., 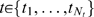. For each time 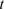, an arbitrary scalar functional quantity 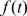 is given which can be computed from the protein coordinates and/or velocities. Note that for the following presentation of FMA, the time 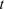 is only an index to label the input structures 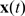 and should not imply that the structures must correspond to a time series. Instead, the structures 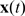 may equally well derive from, e.g., Monte Carlo sampling or from a large ensemble of experimental structures.

#### Maximally correlated motion (MCM)

We seek a normalized collective vector 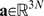 of protein atoms such that the motion along 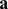 is maximally correlated to the change in the functional quantity 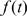. Therefore, the motion along 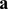 is referred to as ‘maximally correlated motion’ (MCM). The MCM as a function of time 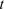 is given by the projection
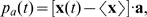
(1)where 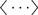 denotes the average over all times 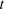.

In the present study, two measures are applied to quantify the correlation between 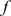 and 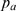. First, Pearson's correlation coefficient defined by
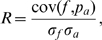
(2)where 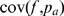 denotes the covariance between 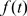 and 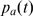, and 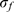 and 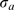 denote the standard deviations of 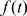 and 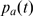, respectively. The Pearson coefficient measures only linear correlation. Second, the mutual information (MI) between 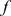 and 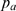 given by [18]

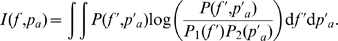
(3)Here, 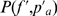 denotes the joint probability distribution of 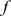 and 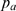, and 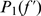 and 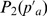 denote the marginal probability distributions of 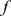 and 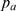, respectively. The MI measures any kind of interdependence between 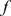 and 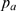, including non-linear and higher order correlation. Note that if (and only if) 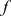 and 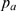 are independent, 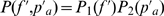 holds, the logarithm in eq. (3) vanishes, and the MI equals to zero. Hence, the MI can be interpreted as the probability weighted deviation from the case of 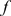 and 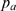 being independent.

#### Reduction of dimensionality

Before optimizing 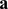 (via maximization of 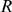 or of the MI), a reduction of the dimensionality of the optimization problem is frequently required. Even when restricting the analysis to a subset of the protein atoms (such as the backbone), the long autocorrelations in protein dynamics may otherwise lead to an overfitted collective vector 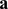. A common procedure to reduce the dimensionality of protein dynamics is principal component analysis (PCA) [10]. PCA allows one to determine a small set of collective vectors with the largest contribution to the mean square fluctuations (MSF) of the atomic coordinates.

For convenience and to clarify the nomenclature we briefly sketch the PCA in the following. Given the 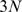 cartesian atomic coordinates 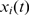 (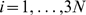), the elements of the covariance matrix 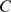 of the atomic positions are given by
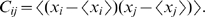
(4)Before computing 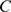, translation and rotation of the entire biomolecule is removed by superimposing the simulation trajectory onto a reference structure. Diagonalization of 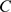 yields a set of 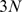 orthonormal eigenvectors 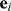 with corresponding eigenvalues 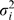. The eigenvectors are typically ordered according to descending eigenvalues and referred to as PCA vectors. The projection 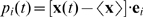 is called 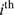 principal component (PC) and quantifies the position of the protein along the 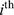 PCA vector.

The MSF of the atoms can be decomposed into contributions from different principal components, 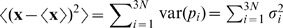, where 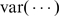 denotes the variance. In protein simulations the first 10–20 PCs (with the largest eigenvalues) often account for a large fraction (80–90%) of the atomic MSF, and higher PCs describe smaller motions such as angle vibrations [10]. Hence, if large protein motions are expected to dominate changes in the 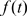, the first few PCA vectors are a reasonable basis set to construct 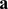.

We stress that PCA vectors are only one possible basis for 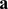. Other possibilities include normal modes or modes derived from full correlation analysis [16],[19]. For some quantities 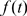 angles in dihedral space or the cartesian coordinates may also provide a useful coordinate system.

#### Maximization of the Pearson coefficient 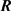


Assuming that 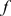 is approximately a linear function of the PCs, the collective vector 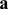 can be derived by maximizing the Pearson coefficient 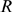 (eq. (2)). We construct 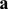 as a linear combination of the first 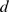 PCA vectors, 
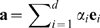
. Here, coefficients 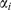 denote the coordinates of 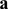 with respect to the basis set 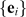.

As shown in the supporting Text S1, a maximum in the absolute value of 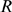 can be found by numerically solving the coupled linear set of equations
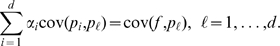
(5)For the present study, 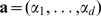 was normalized after computation via eqs. (5). Note that for the maximization of 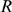, the normalization of 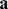 is not strictly necessary because 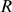 is invariant to the norm of 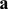.

It is instructive to note that maximizing 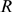 provides a quantitative model for 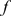 as a function of the PCs 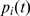. A model for 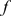 allows one to predict 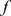 from a given protein structure, and, in turn, propose new structures that generate a particular value of 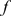 (i.e., a specific functional state). Let
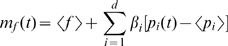
(6)denote the model for 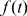. The 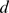 parameters 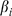 are fitted to the data 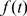 by minimizing the mean square deviation (MSD) between 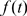 and its model 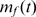, i.e., 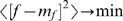. In multivariate regression analysis this approach is referred to as ‘ordinary least square estimation’ [20]. The minimum of 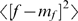 with respect to the parameters 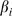 is found by solving the coupled set of linear equations
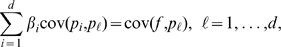
(7)as shown in Text S1. By comparison to eqs. (5), the 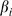 are identical to 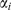 with the exception that the 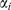 may be scaled by an arbitrary factor without changing 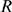. Hence, 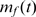 can be rewritten in terms of 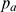 via 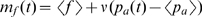, where 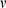 is a constant.

#### Maximization of the mutual information (MI)

If 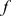 depends non-linearly on atomic positions, the Pearson coefficient might be an insufficient measure to detect correlation between 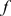 and 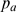. In such cases, we apply the MI as correlation measure because it can detect any kind of interdependence. Optimizing the MI is computationally more demanding as compared to the optimization of 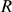. The methodological details for the MI optimization are described in the section ‘Iterative optimization of the mutual information’.

Maximizing the MI yields the collective vector 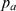 that, by construction, provides as much information on 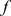 as possible. Because the functional relation between 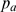 and 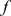 can be arbitrary, optimizing the MI does not directly provide a quantitative model for 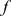 (as in the case of optimizing 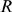, compare previous paragraph). A natural approach to nevertheless yield a model for 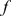 is to fit a general curve 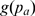 (such as a spline [21] or a polynomial) to the 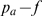 data points. Given a protein structure 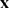, this model allows one to predict the quantity 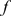 from the structure via 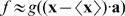.

#### Contributions of principal components to 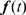


PCA modes have frequently been shown to be important to protein function [8],[10],[22]. However, a functionally important motion may be spread over a number of PCA modes. To understand the protein's function, it is therefore instructive to quantify the influence of different PCA modes on the functional quantity 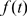, in particular if the PCA modes are related to intuitive motions such as hinge-bending or torsional modes.

Let us first consider the case of a linear model for 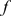 (eq. (6)). Using the linear model for 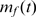 as an approximation for 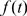 (eq. (6)), the variance of 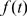 can be approximated by
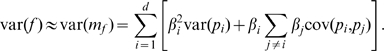
(8)The expression in square brackets is the contribution of the 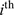 PC to the variance of 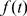. When using the same set of simulation frames for the PCA and for constructing the model 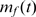, all 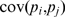 vanish for 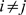 and eq. (8) simplifies to 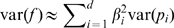.

In case of a non-linear dependence between 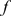 and 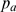, 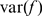 cannot be decomposed into contributions from different PCs. Instead, the variance of 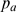 can be written as the right hand side of eq. (8), except for the 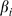 being substituted by the 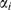. This way, fluctuations of the motion correlated to 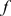 (but not the variance in 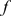 itself) can be decomposed into contributions from different PCs.

#### Ensemble-weighted maximally correlated motion contributing to 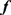


**Figure 1:**
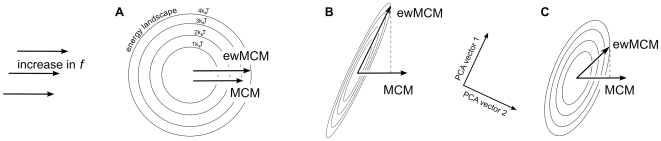
On the difference between the maximally correlated motion (MCM) and the ensemble-weighted MCM (ewMCM) contributing to the functional quantity *f*. (A–C) Irrespective of the energy landscape (thin lines), the MCM (along 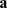) is parallel to the direction with increasing 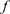 (here, to the right). In contrast, the ewMCM is highly dependent on the energy landscape. (A) If no direction is favored by the energy landscape, the ewMCM is parallel to the MCM. (B) If one direction (PCA vector 1) is highly favored over another direction (PCA vector 2), a displacement along the MCM is mainly accomplished through a motion along the PCA vector 1. Therefore, the ewMCM is nearly parallel to PCA vector 1 in this case. (C) An intermediate situation between the extreme cases (A) and (B). In that case, both PCA vectors 1 and 2 contribute to the ewMCM.

The MCM along the collective vector 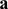 displays the largest correlation to 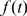 as measured from 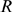 or from the MI. However, due to the protein's energy landscape, the motion parallel to 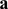 may be severely restricted. This fact is schematically illustrated in Fig. 1. Let us assume that the functional quantity 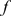 increases to the right in Fig. 1. Then, irrespective of the energy landscape (thin lines in Fig. 1A–C), the MCM is parallel to the direction of increasing 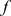. However, if the energy landscape restricts the motion parallel to the MCM (Fig. 1B), a displacement along the MCM will actually occur through a motion which is non-parallel to the MCM, but which is in accordance to the energy landscape. Such motions would have a substantial projection on the MCM, but are not identical to the MCM.

In addition to the MCM, we therefore seek the *most probable* collective motion that accomplishes a specific displacement along the MCM. We apply the input structure ensemble to estimate the most probable motion, and refer to that motion as ‘ensemble-weighted maximally correlated motion’ (ewMCM). The ewMCM is shown as black arrows in Fig. 1 for three different hypothetical energy landscapes. Note that the ewMCM is pointing in the direction that accomplishes a displacement along the MCM with a motion of smallest energy increase (i.e., with the largest probability). If the energy landscape does not favor any direction (Fig. 1A), the ewMCM is parallel to the MCM. In contrast, if the energy landscape highly favors motions in one direction over motions in another direction (Fig. 1B), the ewMCM may strongly deviate from the MCM. An intermediate situation is shown in Fig. 1C. It should be emphasized that the ewMCM is the collective motion *in the given input ensemble* which accomplishes the displacement along the MCM. In case of limited sampling, a second input ensemble may accomplish a displacement along the MCM through a different ewMCM, rendering the ewMCM highly dependent on the input ensemble. This characteristic of the ewMCM motivates the term ‘ensemble-weighted’.

Let us assume that the MCM 
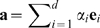
 has been optimized such that 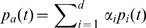 is maximally correlated to 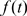, as measured from 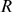 or from the MI (see above). Thus, the set of 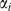 are fixed in the following, and 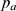 quantifies the functional state of the protein. In the input ensemble, the collective variable 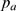 varies between its minimum 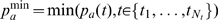 and its maximum 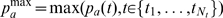. To define the ewMCM, we choose an arbitrary but fixed value for 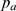, denoted 
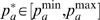
, between its extremes 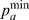 and 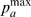. As ewMCM we consider the *most probable* collective displacement 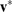 (from the average structure 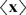) that generates the functional state 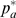,
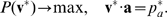
(9)Here, 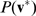 denotes the probability of the collective atomic displacement 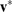. In the following we restrict the ewMCM 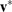 to the subspace spanned by the first 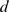 PCA vectors 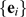. Then, the ewMCM can be expressed as 
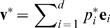
 and the condition (9) can be rewritten as
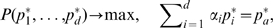
(10)where 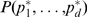 denotes the probability for a particular set of PCs 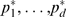.

The 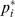 were estimated as follows. First, to simplify the nomenclature, let assume the mutual covariances between the PCs to equal zero. Then, 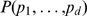 can be approximated via
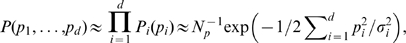
(11)where 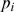 denotes the marginal probability distribution of the 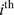 PC, and 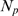 is a normalization constant. Here, the 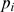 were assumed to be mutually independent and normally distributed. (If the PCs were constructed from a different set of frames than the frames used for the FMA, the covariances between different PCs may not vanish, rendering the assumption 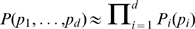 a poor approximation. In that case we switch to new coordinates 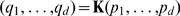 with zero mutual covariances. Here, the transformation matrix 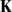 is computed from a PCA on the 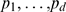.) Using the approximation in eq. (11), the maximization of 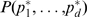 with the constraint 
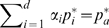
 is straight-forward using Lagrange multipliers. The calculation yields
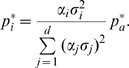
(12)Note that the components 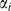 of the MCM are weighted by the variance 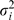 of 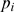 of the given ensemble, further justifying the term ‘ensemble-weighted’. To visualize the ewMCM for the given ensemble, successively increasing values for 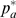 can be chosen between, e.g., 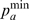 and 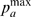. For each value 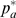, eq. (12) provides a set of PCs 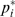 and, hence, a structure 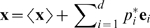. The structures can be depicted in common molecular visualization software.

### Cross-Validation

MD simulations of proteins can be subject to long autocorrelations. The maximization of 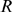 or MI can lead to overfitting if too many free parameters 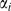 are used in the optimization. It is therefore essential to cross-validate the derived model for 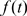 with an independent set of simulation frames. A convenient approach to cross-validate the optimization is to divide the simulation into frames for model building and for cross-validation. Accordingly, 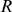 or MI is optimized applying the model building set only, yielding a correlation 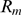 between data and model. Subsequently, the derived model is validated by predicting 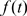 from the derived model using the cross-validation set only, yielding a correlation 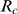. Note that in the present context the term ‘predict’ should *not* imply any prediction into the future. Instead, the exact (or true) 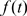 as computed from all atomic coordinates is compared with 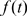 as computed from the model, making only use of the functional collective coordinate 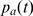. Hence, we apply the term ‘predict’ as common in, e.g., the pattern recognition literature [23]. Using this approach, overfitting is indicated by a substantially smaller 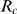 as compared to 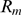.

How many basis vectors 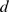 should be used to construct 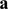
? The optimal number 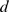 will highly depend on the simulation system and the observable 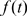. The reasonable choice for 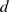 can be identified by plotting 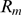 and 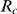 as a function of 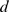. As long as no overfitting occurs, 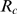 increases with 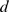, indicating an improvement of the model. As soon as 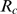 decreases with 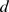 or becomes substantially smaller than 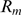, the model is overfitted.

### Iterative Optimization of the Mutual Information

The collective vector 
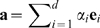
 that yields the largest MI between 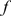 and 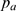 must be optimized iteratively. The optimization procedure for the MI was implemented as follows. The initial guess 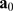 for 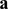 is generated randomly, or corresponding to the optimized Pearson coefficient (eqs. (5)). Subsequently, 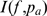 is optimized via a sequence of rotations 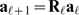 which effect only three coefficients 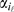, 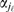, and 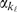. Hence,
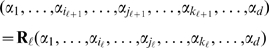
(13)where 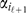, 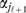, 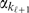 are derived from the 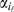, 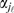, 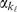 by a three-dimensional (3D) rotation 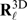,
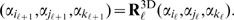
(14)For each optimization step, the 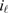, 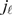, and 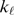 are randomly chosen from the 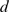 dimensions.

Each 3D rotation matrix 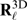 is chosen such that it optimizes the MI in the 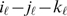 subspace. To optimize 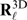, approximately 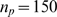 points are uniformly distributed on a unit sphere, each point corresponding to a possible 3D rotation with rotation angles 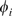 and 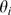 (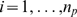). The MI 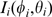 is computed for each of the 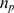 rotations. Subsequently, a set of spherical harmonics (up to order 5) is fitted to the 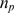 discrete 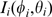, yielding a continuous and smoothed estimate of the MI as a function of the rotation angles 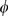 and 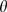. The fit was implemented as a least-square fit using singular value decomposition. Eventually, the function 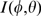 is optimized by Powell's method [24], yielding the best 3D rotation matrix 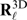 of the 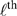 optimization step. The 3D rotations are repeated in different 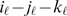 subspaces until convergence.

The MI 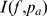 is estimated from the discrete data sets by a binning procedure. Accordingly, the probability distributions 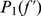 and 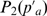 are approximated by counting the occupancies 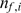 and 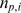 of 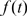 and 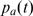, respectively, in bins 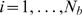. For the present study, the number of bins 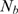 was found to have a minor effect on the results. A reasonable choice was 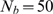. Likewise, 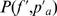 are approximated by a two-dimensional (2D) binning, yielding the 2D occupancy 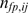. The MI is estimated via
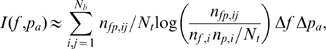
(15)where 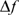 and 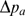 denote the bin widths of 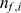 and 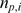, respectively. Note that the technique proposed here does not require an estimate of the absolute MI, but only of the relative change in MI due to a rotation 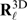. Sophisticated and computationally demanding methods such as kernel density estimates are therefore unnecessary.

### Simulation Setup

The Fs_21_ helix (originally introduced by Lockhart *et al.* [25], Sequence Ace-A_5_[AAARA]_3_A-NH_2_) was modeled with PyMol [26]. The structures of T4 lysozyme (T4L) and leucine-binding protein (LBP) were taken from the protein data bank (PDB codes 256L [27] and 1USG [28], respectively). Likewise, the first structure in the NMR ensemble derived by Neidigh *et al.* (PDB code 1L2Y [29]) was used as initial structure for the simulations of Trp-cage. The Fs_21_, T4L, Trp-cage, and LBP structures were placed into dodecahedral simulation boxes and solvated with 8828, 8479, 3042, and 17581 explicit water molecules, respectively. All simulation systems were neutralized by adding chloride ions. In addition, 150 mM sodium chloride was added to the Trp-cage and LBP systems.

The Fs_21_ helix and LBP were simulated with the AMBER03 [30] force field and the TIP3P water model was applied [31]. Ion parameters were taken from Smith *et al.* [32]. Trp-Cage and T4 lysozyme were simulated with the OPLS all-atom force field [33] and the TIP4P water model [34]. All simulations were carried out using the GROMACS simulation software [35],[36]. Electrostatic interactions were calculated at every step with the particle-mesh Ewald method [37],[38]. Short-range repulsive and attractive dispersion interactions were described by a Lennard-Jones potential, which was cut off at 1.0 nm (0.8 nm for the AMBER03 simulation). The SETTLE [39] algorithm was used to constrain bond lengths and angles of water molecules, and LINCS [40] was used to constrain the peptide bond lengths, allowing a time step of 2 fs.

The temperature in the Fs_21_ and T4L simulations was kept constant by weakly (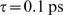) coupling the system to a temperature bath [41] of 300 K. Likewise, the pressure was kept constant by weakly coupling the system to a pressure bath of 1bar with a coupling constant 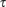 of 1 ps. The LBP and Trp-cage systems were coupled to a Nosé-Hoover thermostat [42],[43] (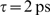) at 300 K and 400 K (to trigger unfolding), respectively, and the pressure was kept at 1 bar using the Parrinello-Rahman pressure coupling scheme [44] (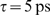).

The Fs_21_ helix was simulated for 250 ns and the structure was written to the hard disk every picosecond. During the simulation the helix partially unfolded and refolded for a number of times. Because we chose to consider collective motions of an intact helix only, the secondary structure was determined for every frame with DSSP [45] and only frames with a complete helix were used for further analysis. Approximately 53.000 such frames were found which were combined into one ‘trajectory’ of 53 ns with time step 1 ps. The lysozyme simulation system was simulated for 460 ns, and the LBP system for 100 ns. The Trp-cage protein was simulated 8 times for 40 ns with different initial velocities. The volume of the catalytic cleft of T4L was estimated as explained and illustrated in supporting Figure S1.

## Results

In the following we apply FMA on four biological examples, and demonstrate how quite different functional quantities 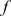 can be related to collective protein motions. In the first three examples of increasing complexity (Fs_21_ helix, T4 lysozyme, and Trp-cage) the Pearson coefficient turned out to be sufficient to detect correlations between the respective functional quantity and collective motions. With the final example (leucine-binding protein) we demonstrate how the MI can elucidate correlation in cases where the Pearson coefficient fails.

As a first and trivial example we analyze collective motions related to the end-to-end distance of the Fs_21_ helix. The example (including figures) is presented in supporting Text S2 and illustrates the application of FMA in some detail. Because the PCA vectors correspond to the harmonic modes of a simple helical spring, the decomposition of the end-to-end distance into contributions from different PCA modes is particularly instructive in this example.

### Collective Motions of T4 Lysozyme Involved in Enzymatic Activity

Domain motions of hen lysozyme have been proposed more than 30 years ago [46],[47]. Likewise, domain motions in T4 lysozyme (T4L) have been studied intensively by X-ray crystallography [27], [48]–[50], site-directed spin labeling [51], as well as by theoretical approaches such as normal mode analysis, MD and PCA [47], [52]–[54].

**Figure 2:**
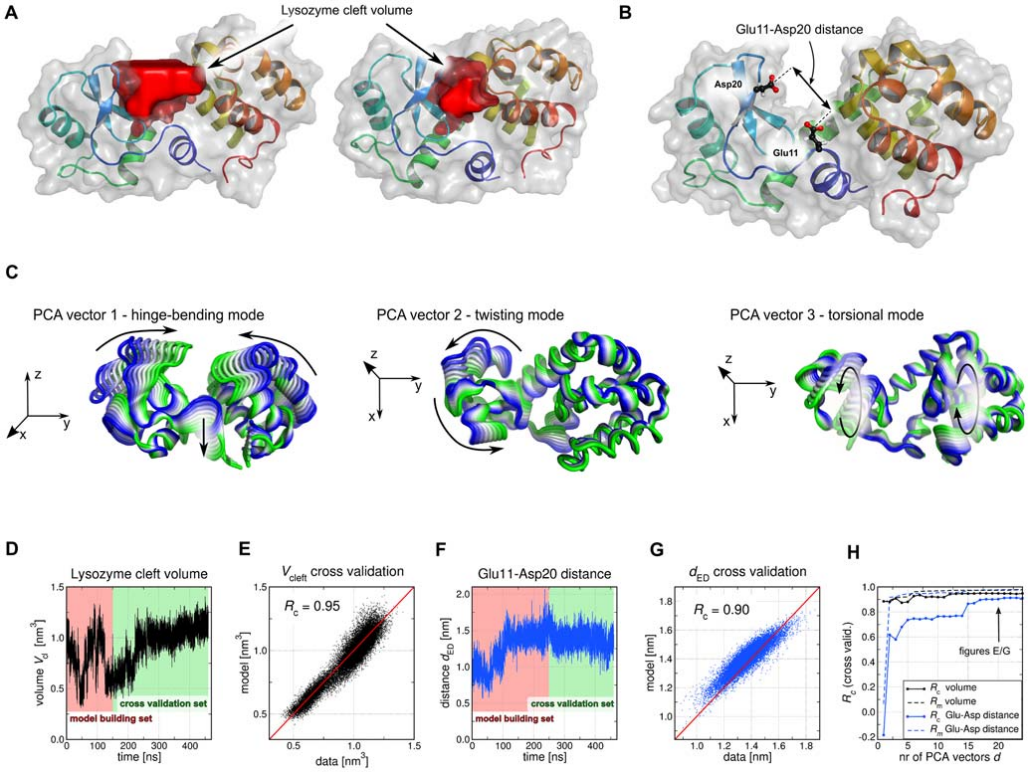
Functional mode analysis of catalytic cleft volume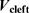 and Glu11-Asp20 distance 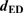 of T4 lysozyme (T4L). (A/B) T4L in cartoon and surface representation. The catalytic cleft is shown as red surface (A), and Glu11 and Asp20 are depicted in ball-and-stick representation (B). (C) The motions along the first three PCA vectors. (D) 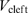, and (F), 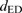 versus the simulation time (black and blue curves, respectively). The first 180 ns (250 ns for 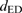) were used as model building sets (red background), the remaining simulation frames as cross-validation sets (green background). (E/G) Scatter plots of the data versus the model using the cross-validation sets only. (H) Correlations 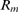 and 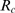 for 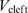 (black curves) and 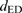 (blue curves), presented as a function of the number of principal components 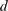 used during the optimization.

Here we demonstrate how FMA can be applied to determine the collective motions which are putatively involved in the enzymatic activity of T4L. Two functional quantities 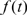 related to the enzymatic activity are considered for the analysis. (i) The volume of the catalytic cleft 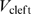, highlighted as red surface in Fig. 2A, and (ii) the distance 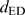 between the carboxyl groups of the catalytic residues Asp20 and Glu11 (Fig. 2B). The volume 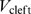 is biologically significant because opening and closure of the cleft is expected to be involved in substrate binding and product release. The distance 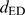 is a direct measure of the geometry of the catalytic site. According to the textbook mechanism proposed by Phillips [55], Glu11 protonates the glycosidic oxygen while Asp20 stabilizes the produced oxocarbenium ion intermediate. Hence, the carboxyl groups of Glu11 and Asp20 must simultaneously arrange closely to the glycosidic bond. 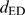 is therefore an easily assessable observable that probes enzymatically active configurations. The distance between the carboxyl groups was measured as the distance between the 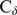 atom of Glu11 and the 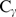 atom of Asp20.

In the following, the results of the FMA of 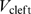 and 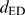 are presented in a relatively compact fashion. For a more detailed presentation of FMA we refer to the illustrative 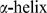 example in supporting Text S2. In a first step, the basis set 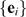 was derived by a PCA on the backbone atoms, using the 460-ns T4L simulation. The first 20 PCA vectors were used as basis set for the FMA. The motions along the first three PCA vectors are shown in Fig. 2C. The first PCA vector corresponds to the well-studied hinge-bending mode of T4L [46],[51], and the second to a twisting mode, mainly characterized by a rotation of the smaller (N-terminal) lysozyme domain. The third PCA vector corresponds to the torsion of the N-terminal domain with respect to the C-terminal domain.

In Figures. 2D and 2F, 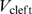 and 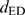 are plotted as a function of simulation time, respectively. The first 180 ns (250 ns for 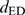) were applied as model building sets (red background), the remaining frames as cross-validation sets (green background). For both 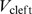 and 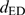, the respective collective vector 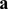 was optimized by maximizing 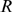, yielding linear models for 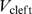 and 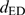. The models are validated in figs. 2E and 2G, showing scatter plots between simulation data and models using the respective cross-validation sets only. Strong cross-validated correlation (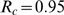 and 0.90, respectively) between model and data was found, confirming the validity of the models. (The corresponding scatter plots using the model building sets are presented in Figure S3.) Note that side chain fluctuations of Glu11 and Asp20 cannot be modeled from backbone PCA modes. Yet the derived model for 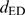 favorably correlates with the data (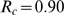), indicating that side chain fluctuations have only a minor effect on 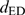. Figure 2H shows the 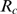 values for 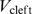 and 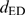 as black and blue curves, respectively, as a function of the number of PCA vectors 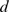 used in the FMA. Apparently, the first PC already provides a good model for 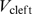 (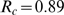). In contrast, at least 15 PCs are required to construct a good model (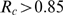) for 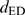.

**Figure 3:**
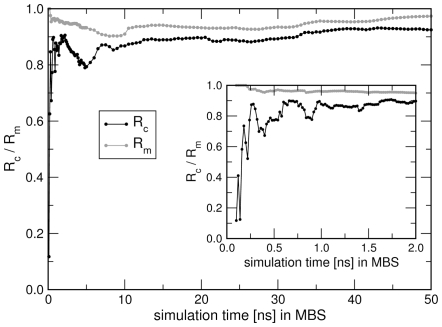
Convergence of FMA of the lysozyme cleft volume 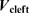. 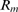
 (gray) and 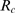 (black curve) as a function of simulation time in the model building set (MBS). For each data point in the figure, all remaining frames from the 460-ns trajectory were used as cross validation set, and the first 20 PCA vectors were applied as the basis set to construct 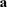. The inset displays the simulation time in the MBS is in a detailed scale. Applying approx. 10 ns of simulation as MBS is sufficient to yield a reasonable model (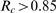) for the remining frames. Applying less than 0.5 ns as MBS yields an highly overfitted model.

The convergence of FMA of 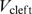 with the number of frames in the model building set is analyzed in Fig. 3. The figure plots 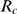 and 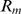 between the 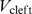 data and 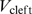 model as a function of the simulation time in the model building set. All remaining frames of the 460-ns trajectory were applied for cross validation. Remarkably, using only 10 ns for model building yields a reasonable model (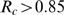) for the remaining frames. In contrast, using less than 0.5 ns for model building yields a highly overfitted model, as visible from the large 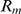 as compared to 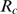. The related analysis for the FMA of 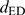 is presented in Fig. S2B.

**Figure 4:**
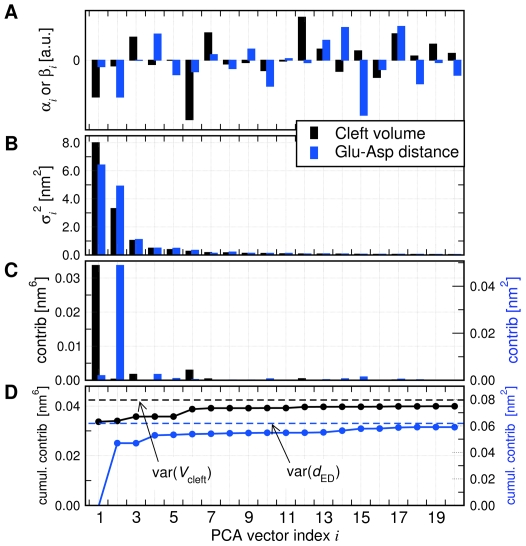
Collective vector ‘
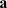
’ related to the lysozyme cleft volume 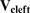 (black) and to the distance 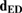 between Glu11 and Asp20 (blue). (A) components 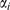 of 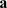 with respect to the PCA vectors 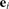. (B) Variances 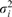 of the principal components (PCs), (C) contribution of the 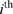 PC to the variance of the model, and (D) the cumulative contribution of principal component 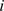 to the variance of the model. The dashed lines indicates the variances of 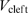 and 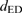 during the simulation.

Figure 4 presents the collective vectors related to 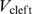 and 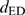, as well as the contributions of different PCs to 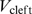 and 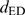. The results for 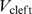 are presented as black bars and curves, the results for 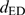 in blue. The coefficients 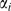 of 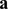 (or 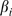 of the linear model, eq. (6)), are shown in Fig. 4A. Note that 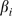 quantifies the effect of the 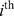 PC on 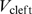 (or 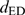) *per nanometer displacement* in PCA space. Because the variance of the PCs rapidly decay with increasing PC index 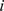 (Fig. 4B), only the first PCs substantially contribute to the variances of 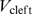 and 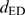 (Fig. 4C). Remarkably, the first PC almost completely accounts for 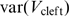, whereas the second PC accounts for 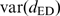. Figure 4D presents the cumulative contributions of the PCs to 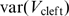 and 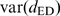 as derived from the respective models as solid curves, and the variances of 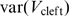 and 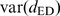 as dashed curves. The plot confirms that the models indeed account for a large fraction the variances of 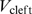 and 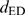, respectively.

Which are the ewMCMs contributing to 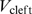 and 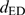
? Applying eq. (12) shows that the ewMCMs contributing to 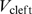 and 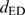 are highly related to PCA vectors 1 and 2, respectively, a finding in agreement to Fig. 4C. For the illustration of these motions we therefore refer to the PCA vectors depicted in Fig. 2C. In addition, the MCM and the ewMCM for both 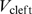 and 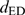 are shown in supporting videos S1 and S2.

Taken together, the FMA provides a comprehensive picture of the collective motions involved in the catalytic activity of T4L. The hinge-bending mode (Fig. 2C) dominates closing and opening of the catalytic cleft, presumably facilitating substrate binding and release. Surprisingly, this mode leaves the active site geometry virtually unaffected. In contrast, the twisting mode dominates the distance 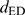 between the carboxyl groups of Glu11 and Asp20. Hence, a major collective rotation of the N-terminal domain with respect to the C-terminal domain may be required to position Glu11 and Asp20 into an enzymatically active configuration.

### Initial Unfolding of Trp-Cage

**Figure 5:**
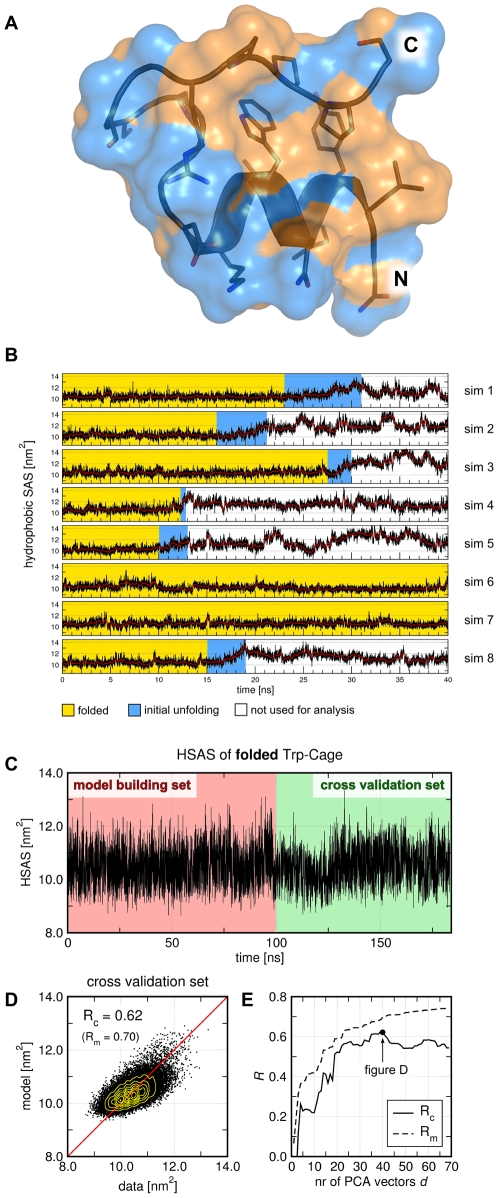
Functional mode analysis of the hydrophobic solvent-accessible surface (HSAS) of Trp-cage in the folded state. (A) Trp-cage protein in the folded state, shown in cartoon and surface representation. The HSAS is shown as orange surface, the hydrophilic SAS as blue surface. (B) HSAS during 8 independent simulations (sim 1-8). HSAS (black curves), and to guide the eye, the HSAS smoothed by a moving average (red curves). In simulation frames highlighted by a yellow background, Trp-cage was considered as folded. The initial unfolding events are highlighted by a blue background. (C) HSAS versus simulation time (black curve) combined from all folded states of the 8 Trp-cage simulations (B). The first 100 ns were used as model building set (red background), the remaining 83.3 ns as cross-validation set (green background). (D) Scatter plot of the data versus the model using the cross-validation set. (E) Correlations 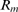 and 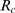 of the model building and cross-validation sets, respectively, versus the number of PCA vectors 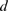 used during the optimization.

Trp-cage is a 20-residue miniprotein designed by Neidigh *et al.* [29]. With a folding time of 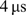 [56] Trp-cage is the fastest folding protein currently known. Trp-cage is characterized by a central tryptophan side chain (Trp6) which is surrounded by an 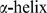 (residues 2–8), a 3_10_-helix (residues 11–14), and a C-terminal polyproline helix (Fig. 5A). Here we use Trp-cage as a model system to demonstrate how FMA can be applied to study the structural determinants underlying the initial unfolding process of a protein. To this end, the hydrophobic solvent-accessible surface area (HSAS) is used to quantify the state of unfolding.

Compared to the distances and volumes considered so far as functional quantities 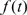, explaining the HSAS by single collective mode is challenging. The HSAS is subject to strong noise and is a non-linear function of the atom coordinates. Only the linear parts of the dependence of the HSAS on the PCs is expected to be successfully captured by the linear model of eq. (6). The non-linear dependence on the PCs (that would have to be described as cross terms of the PCs) will appear as a noisy deviation from the model.

We use FMA to determine the collective motions related to the change in the HSAS, and hence, to the initial unfolding of Trp-cage. Three questions are addressed: (i) To which extent can a model based on a single collective motion explain a highly non-linear quantity such as the HSAS? (ii) Which collective motions increase the HSAS and, hence, represent the initial unfolding of Trp-cage? And (iii), can a model derived only from fluctuations in the folded state predict the HSAS during an unfolding event? To observe multiple unfolding events, eight 40-ns simulations were performed at a temperature of 400 K. The HSAS during the eight simulations is shown in Fig. 5B. In six of the eight simulations, the Trp-cage unfolded after simulation times between 10 and 27.5 ns (blue background in Fig. 5B). In the other two simulations the Trp-cage remained folded for the complete simulation time. From the eight simulations, all frames of the folded Trp-cage (yellow background in Fig. 5B) were combined into one ‘folded trajectory’ of 183.3 ns (916503 frames).

The HSAS of the ‘folded trajectory’ is plotted in Fig. 5C. The first 100 ns were applied as model building set in the FMA (red background), the remaining 83.3 ns as cross-validation set (green background). The basis set for the FMA was taken from a PCA of all heavy atoms (after a least-square fit of the backbone atoms onto the 1L2Y structure). The first 40 PCA vectors were used as basis set. The PCA vectors are not visualized because they do not correspond to easily interpretable motions. From the model building set, a linear model for the HSAS was derived using eqs. (7), and the model was validated using the cross-validation set (Fig. 5D). The correlation between data and model is substantially weaker (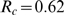, 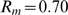, see Fig. S3D) than in the previous examples. As expected, the HSAS *in the folded state* is only partially captured by the linear model. To reach a similar model quality as in the previous examples, additional non-linear cross terms of the PCs would have to be included into the model. Without such additional terms, the deviation from the linear model appears as noise. Analysis of the difference between data and model shows that the noise is normally distributed around zero with a standard deviation of 0.36 nm^2^ (not shown).

To avoid overfitting, 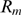 and 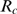 are plotted as a function of the number of PCA vectors 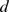 used as basis set (Fig. 5E). Both 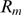 and 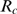 increase up to 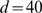, corresponding to an improvement of the model. For 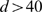, only 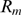 increases, but 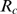 decreases with 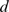. Hence, using more than 40 PCA vectors as basis set would yield an overfitted model.

**Figure 6:**
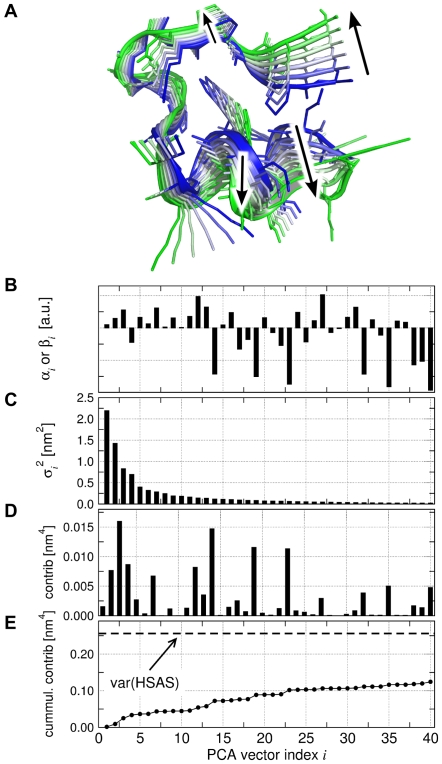
Collective motion related to the increase in hydrophobic solvent-accessible surface (HSAS) of Trp-cage. (A) Cartoon representation of the ensemble-weighted MCM contributing to the increase in HSAS. Side chains are shown as sticks. (B) Eigenvalues 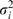 of the principal components (PCs), (C) components 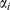 of the collective vector 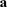 with respect to the PCA vectors 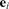. (D) Contribution and (E) cumulative contribution of the 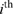 PC to the variance of the model. The dashed line indicates the variance 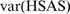 of the HSAS during the simulation.

The ewMCM related to the increase in HSAS is visualized in Fig. 6A. The motion is mainly characterized by a lift-off motion of the polyproline helix with respect to the 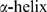. The ewMCM and the MCM along 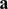 are also shown in video S3. The components 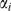 of 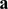 (or 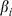 of the model) are shown in Fig. 6B, and the variances of the PCs in Fig. 6C. As visible from Fig. 6D, many PCs contribute to the variance of the HSAS 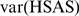. Remarkably, PCs with larger index 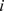 substantially contribute the the HSAS, although they hardly contribute to the MSF of the atom positions (compare Fig. 6C). The cumulative contribution of the PCs to 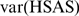 (Fig. 6E) indicates that approximately 50% of the variance (corresponding to 70% of the standard deviation) of the HSAS in the folded state are explained by the linear model.

**Figure 7:**
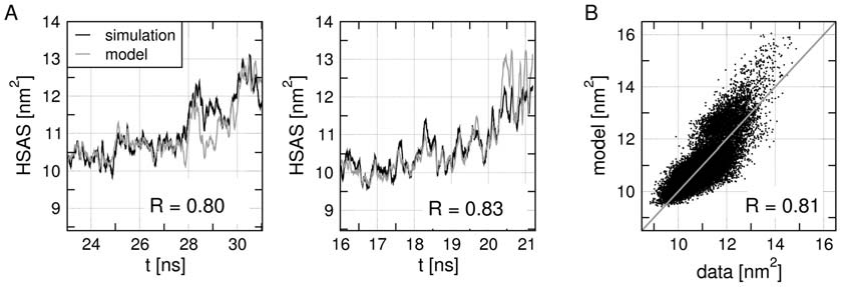
Predictive power of HSAS model during the initial unfolding events. (A) HSAS (black) during unfolding events of Trp-cage simulations 1 and 2 and the prediction by the HSAS model (gray). The correlations 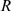 between model and data are printed as insets. To facilitate the comparison between data and model in these plots, all HSAS curves were slightly smoothed by running averages. The 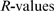 were computed from the non-smoothed data (not shown). The HSAS and the prediction of all six unfolding events are shown in the supporting material. (B) Scatter plot of HSAS data versus model as collected from all six unfolding events.

Can the model for the HSAS derived from fluctuations *in the folded state* predict the HSAS during unfolding? To assess this particularly rigorous test for the validity of the model, the HSAS during six independent unfolding events was monitored (blue background in Fig. 5B). Figure 7A displays two examples for the HSAS during unfolding events as black curves, and the HSAS predicted by the model as gray curves. The corresponding plots for all six unfolding events are shown in Fig. S4. Good agreement is found with correlation coefficients 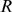 between data and model in the range of 0.72 to 0.88 (insets in Fig. 7 and S4). The respective scatter plot of HSAS data versus model as combined from all six unfolding events is shown in Fig. 7B. Reasonable agreement (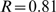) between data and model is found. Note that such unfolding events are not present the model building set. Hence, the model derived only from the folded state displays predictive power during a process (initial unfolding) which did not occur in the model building set. Noteworthy, favorable correlation between data and model during unfolding can only be achieved if the collective unfolding modes are at least partially sampled in the folded state fluctuation. If unfolding modes do not sufficiently fluctuate in the folded state, no correlation between such modes and the HSAS can be detected. Figures 7 and S4 show, however, that folded state fluctuations in this case are sufficient to construct the HSAS model (and hence, the functional mode) that holds during initial unfolding.

### A Non-Linear Example: RMSD of Leucine-Binding Protein

**Figure 8:**
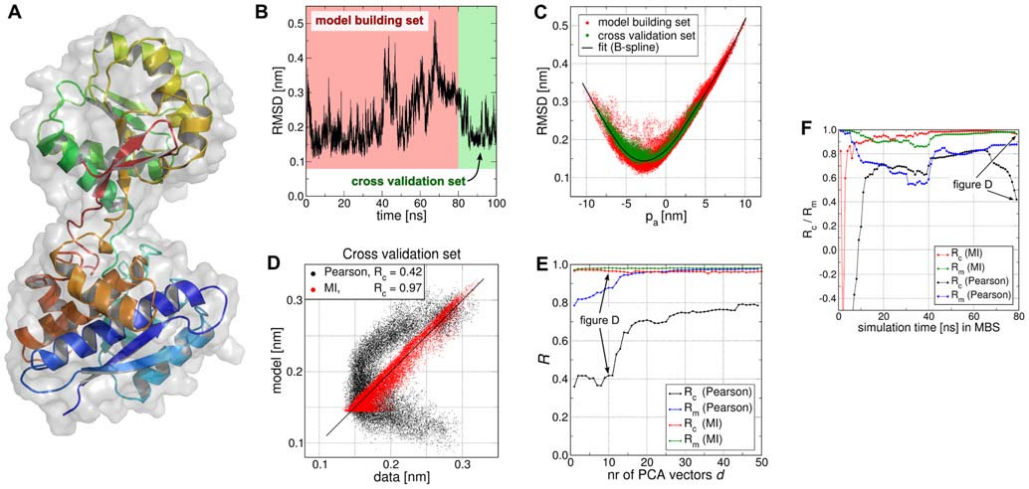
Functional mode analysis of the RMSD of leucine-binding protein (LBP) with respect to its apo structure. (A) Apo structure of LBP in cartoon and surface representation. (B) RMSD with respect to the apo structure versus simulation time. Model building and cross-validation sets are highlighted by red and green background, respectively. (C) RMSD versus the collective coordinate 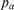 optimized via mutual information (MI). Model building set (red dots), cross-validation set (green dots), and spline fitted to the model building set (black curve). (D) Scatter plot of cross-validation set showing model versus data. Optimizing MI yields favorable correlation (red dots, 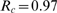), optimizing the Pearson coefficient only poor correlation (black dots, 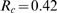). (E) Correlations 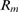 and 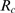 of the model building and cross-validation sets, respectively, versus the number of PCA vectors 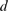 used during the optimization. MI optimization (green/red curves) is compared to Pearson optimization (blue/black curves). (F) Convergence of FMA with simulation time in the model building set (MBS). 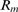 and 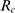 (from Pearson and MI optimization, compare legend) are showns as a function of simulation time in the MBS using 10 PCA vectors as basis set. All remaining frames of the 100-ns trajectory were applied as cross validation set.

As an example for a non-linear correlation between a functional quantity 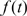 and collective motions we consider the root mean square deviation (RMSD) of the backbone atoms of l-leucine-binding protein (LBP). LBP is a two-domain transport protein (Fig. 8A) that is subject to a large hinge motion (0.7 nm RMSD) upon ligand binding [28],[57]. The RMSD was computed with respect to the (apo) crystal structure. The RMSD increases as the protein deviates from the reference, irrespective of the direction of the collective motion. Hence, it can not be explained in terms of a linear function of a collective coordinate. Because the RMSD is frequently assessed in MD studies, we use it as a model quantity to demonstrate the use of mutual information (MI) in FMA.

The RMSD is plotted in Fig. 8B. The collective vector 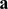 was optimized such that 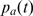 displays the maximal MI to the RMSD. To this end, the first 80 ns of the simulation were applied as model building set, omitting the first nanosecond for equilibration (red background in Fig. 8B), and the remaining frames were applied as cross-validation set (green background). The first 10 PCA vectors from a PCA of the backbone atoms were used as basis set for 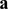 (not shown). Figure 8C presents the RMSD versus the optimized collective coordinate 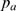 for the model building set (red dots) and the cross-validation set (green dots). As expected, the RMSD and 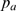 are substantially correlated, and the correlation is highly non-linear as visible from the non-linear 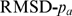 point cloud. The non-linear model for the RMSD was constructed by fitting a cubic B-spline (black curve in Fig. 8C) to the 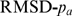 points of the model building set. Using this model, the RMSD of the cross-validation set was predicted and compared to the RMSD from the simulation (red dots in Fig. 8D). Excellent agreement (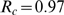) between data and model is found.

For comparison, we tried to derive a linear model for RMSD using the Pearson coefficient 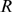 as correlation measure. However, this linear model has little predictive power as visible from model-versus-data scatter plot of the cross-validation set (black dots in Fig. 8D, 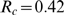). The failure of 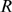 to detect the correlation between RMSD and a single collective motion is also apparent from Fig. 8E which plots 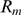 and 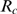 as a function of the number of PCA vectors 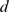 used as basis set. Irrespective of 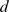, 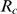 (black curve) is substantially smaller than 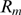 (blue curve), indicating overfitting. In this example, using more than 10 PCA vectors as basis set would increase 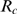, but 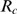 remains much smaller than 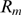. Note that the model derived using the MI as correlation measure displays excellent correlation between data and model for both model building and cross-validation sets (green and red curves in Fig. 8E, respectively).

**Figure 9:**
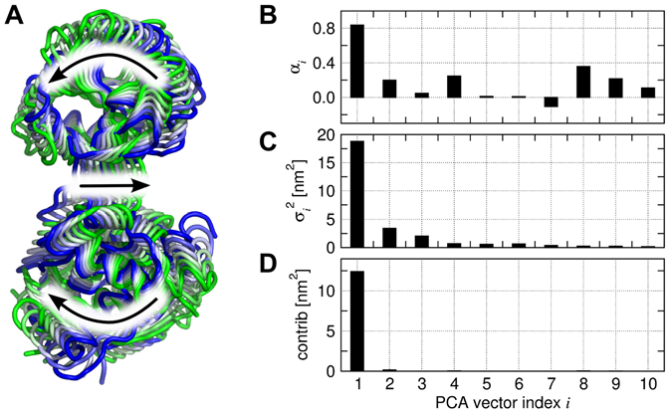
Collective motion related to the increase in RMSD of leucine-binding protein (LBP). (A) Backbone representation of the ensemble-weighted MCM motion contributing to the increase in RMSD. (B) components 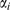 of the collective vector 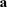 with respect to the PCA vectors 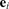. (C) Eigenvalues 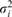 of the principal components (PCs), (D) Contribution of the 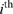 PC to the variance of the collective coordinate 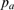.

Figure 9F presents the analysis of the convergence of FMA with the number of frames in the model building set by plotting 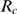 and 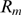 as a function of simulation time in the model building set. All remaining frames of the 100-ns trajectory were applied for cross validation, and the first 10 PCA vectors were used as basis set. When optimizing the MI (red and green curves), a model building set of 11 ns is sufficient to derive a good model (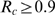) for the remaining simulation. In contrast, using less than 5 ns for model building may yield a highly overfitted model, as visible from a small (or even negative) 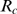 (red curve). When optimizing the Pearson coefficient (black and blue curves), the model quality as measured from 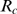 is substantially poorer as compared to the model optimized via MI. In addition, 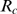 is highly depdendent on the length of the model building set, further emphasizing that the the Pearson coefficient is an unsuitable measure to assess correlation between the RMSD and a single collective mode.

The ewMCM that effects the optimized collective coordinate 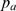 (and hence, the RMSD) is visualized in Fig. 9A. The motion is characterized by a large hinge of the two domains with respect to each other. The collective motion is decomposed into the PCs in figs. 9B–D. The coordinates 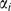 of 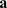 are sown in Fig. 9B and the variances of the PCs in Fig. 9C. Figure 9D displays the contributions of the PCs to the variance of 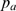, indicating that the first PC almost completely accounts for the variance of 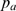. This finding is expected because the first PC is constructed such that it accounts for the largest possible fraction of the RMSD. Hence, Fig. 9D may be considered as a further validation of the technique.

## Discussion

We have presented a novel analysis technique termed functional mode analysis (FMA) to systematically identify functionally relevant collective motions in proteins dynamics. Given an arbitrary quantity 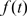 considered relevant to the function of the protein of interest, the approach extracts the linear collective motion which is maximally correlated to 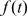. We have used two different measures to quantify the correlation between 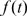 and the collective motion. (i), the Pearson coefficient which measures linear correlation only, and (ii), the mutual information (MI) which can assess any kind of correlation including non-linear and higher order correlation. The ‘maximally correlated motion’ (MCM) must be distinguished from the ‘ensemble-weighted MCM’ (ewMCM) that –based on the sampling in the input ensemble– has the largest likelihood to contribute to large fluctuations in 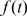. Numerous proteins accomplish their biological function by structural transitions such as hinge motions, domain rotations, or side chain reorientations [2]. For many proteins it is far from obvious how functionally relevant quantities are related to such collective atomic motions. In such cases, the proposed technique is expected to elucidate relations between collective motions (i.e. protein dynamics) and protein function. Moreover, the MCM or the ewMCM can be enhanced or steered during a follow-up simulation, allowing one to trigger functional transitions or to enhance the sampling of rare functional events [58],[59]. Alternatively, the MCM can be biased to compute free energy differences between different functional states (using, e.g., umbrella sampling).

The success of the technique rests on two prerequisites: (i) The collective motion 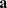 must be representable by a linear combination of the chosen basis set 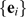. At the same time, the basis set should not be too large to avoid overfitting. (ii) Sufficient (linear or non-linear) correlation between 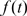 and the single collective motion must be detectable. Future efforts will focus on ameliorating the first condition.

Optimization of the collective vector 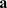 via the Pearson coefficient is closely related to the construction of a model for 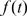 as a linear function of a set of given collective coordinates. When using MI as correlation measure, a non-linear model for 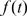 can be constructed from a general set of functions (such as splines). The given collective coordinates used as basis set may correspond to motions along PCA vectors, normal modes, or to motions in any other useful coordinate system such as rotations of dihedral angles. If these given coordinates have an intuitive meaning (such as the hinge-bending mode in T4L), the derived model can quantify the contributions of intuitive collective motions to the variance of 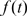, and hence, provide a functional interpretation of the collective dynamics.

The source code of an FMA implementation is available on the authors' web site http://www.mpibpc.mpg.de/groups/de_groot/software.html.

## Supporting Information

Text S1 Text S1
(0.06 MB PDF)

Text S2 FMA of the end-to-end distance of an α-helix
(1.92 MB PDF)

Figure S1 Estimation of the volume of the lysozyme catalytic cleft. A block of test atoms with the approximate shape of the binding site was set up by placing the test atoms on a grid of spacing 1 Å (red block in fig. S1A). The block was placed into the catalytic cleft of a reference structure of T4 lysozyme (T4L). Each T4L structure from the simulation trajectory was fitted onto the reference structure using a least square fit on the backbone atoms. Figure S1A shows one example of a fitted structure, together with the block of test atoms. Subsequently, the test atoms which overlapped with the fitted structure were removed with the genbox tool (fig. S1B). Every remaining atom contributed 1 Å^3^ to the cleft volume.
(0.27 MB JPG)

Figure S2 Convergence of FMA with simulation time. (A–C) Correlations *R*_c_ (black curves) and *R*_m_ (red curves) of the cross validation and model building sets, respectively, as a function of the number of frames (or simulation time) in the model building set (MBS). (A) Helix end-to-end distance *L*_h_. Approximately 30 frames are sufficient to construct a reliable model for *L*_h_, as visible from the *R*_c_ curve (compare Text S2). (B) T4 lysozyme Glu11-Asp20 distance *d*_ED_. Approximately 10 ns of simulation are sufficient to yield reasonable models for *V*_cleft_ and *d*_ED_, although the model quality as measured by *R*_c_ may slightly increase when applying more than 40 ns as MBS. (C) Hydrophobic solvent-accessible surface (HSAS) of Trp-cage in the folded state. From the folded states of the 8 Trp-cage simulations (yellow background in Fig. 5B), an increasing fraction (e.g. 20%) was used as model building set, whereas the remaining fraction (e.g. 80%) of the folded states were applied as cross validation set. As visible from the black *R*_c_ curve, applying more than 30% of the simulation hardly improves the prediction for the remaining frames. The respective plots for the lysozyme cleft volume and the RMSD of leucine-binding protein are shown in Figs. 3 and 8F, respectively.
(0.06 MB PNG)

Figure S3 Scatter plots showing model versus data of the model building sets. (A) Helix end-to-end distance, (B) Glu11-Asp20 distance of T4 lysozyme (T4L), (C) cleft volume of T4L, (D) hydrophobic solvent-accessible surface of Trp-cage, and (E) RMSD of backbone atoms of leucine-binding protein (LBP) with respect to its apo structure. Optimization of the mutual information (MI, red dots) yields larger correlation than optimization of Pearson's coefficient (black dots).
(0.26 MB PNG)

Figure S4 Predictive power of the model for the hydrophobic solvent-accessible surface (HSAS) during six initial unfolding events. HSAS (black curves) during unfolding events and the prediction for the HSAS by the model (red curves). Note that the model was derived only from fluctuations in the folded state. The correlation *R* between model and data (printed as insets) lies in the range of 0.72 to 0.88. To facilitate the comparison between data and model in these plots, all HSAS curves were slightly smoothed by running averages. The *R*-values were computed from the non-smoothed data (not shown).
(0.12 MB PNG)

Video S1 Movie showing the MCM (top) and the ewMCM (bottom) related to the increase of cleft volume *V*_cleft_ of T4 lysozyme.
(2.99 MB MPG)

Video S2 Movie showing the MCM (top) and the ewMCM (bottom) related to the increase of the distance *d*_ED_ between Glu11 and Asp20 in T4 lysozyme.
(3.18 MB MPG)

Video S3 Movie showing the MCM (left) and the ewMCM (right) related to the increase in hydrophobic solvent-accessible surface of Trp-cage.
(2.35 MB MPG)
