# Supplementary material for: Detection of Functional Modes in Protein Dynamics
Source: PLoS Comput Biol. 2009 Aug 28;5(8):e1000480. doi: 10.1371/journal.pcbi.1000480 (PMC2721685; doi:10.1371/journal.pcbi.1000480)
Supplement: Text S1 — (0.06 MB PDF) [file pcbi.1000480.s001.pdf]

Text S1

Maximization of the absolute value of R

Here we derive eqs. (5) of the main manuscript that maximize the Pearson coefficient R. Without loss of generality let us assume that the variance of $f(t)$ equals unity. Then, R reads

$$R = \frac{\mathrm{cov}(f, p_a)}{\sigma_a} = \frac{\sum_{i=1}^{d} \alpha_i \, \mathrm{cov}(f, p_i)}{\left[\sum_{i,j=1}^{d} \alpha_i \alpha_j \, \mathrm{cov}(p_i, p_j)\right]^{1/2}} \tag{1}$$

Note that R is invariant to a scaling of all coefficients α_i by a constant and hence, $\sum_i \alpha_i^2$ must not be treated as a constraint for the optimization. The maximum of the absolute value of R can be found by maximizing R^2. For compact notation, the abbreviations $c_i := \mathrm{cov}(f, p_i)$ and $\gamma_{ij} := \mathrm{cov}(p_i, p_j)$ are introduced. Then, the square of the correlation coefficient reads $R^2 = \left[\sum_{i=1}^{d} \alpha_i c_i\right]^2 / \sum_{i,j=1}^{d} \alpha_i \alpha_j \gamma_{ij}$, and its derivate with respect to α_ℓ is

$$\frac{\partial [R^2]}{\partial \alpha_\ell} = \frac{2 \sum_i \alpha_i c_i}{\left(\sum_{ij} \alpha_i \alpha_j \gamma_{ij}\right)^2} \left[c_\ell \left(\sum_{ij} \alpha_i \alpha_j \gamma_{ij} \right) - \left(\sum_i \alpha_i c_i \right) \left(\sum_i \alpha_i \gamma_{i\ell} \right) \right]. \tag{2}$$

R^2 is maximized by setting all d derivatives of R^2 to equal zero. $\partial[R^2]/\partial\alpha_\ell = 0$ is satisfied for all ℓ if the α_i obey

$$\kappa \sum_i \alpha_i \gamma_{i\ell} = c_\ell, \quad \ell = 1, \ldots, d. \tag{3}$$

Here, κ is an arbitrary constant which can be used to normalize the collective vector $\mathbf{a}$ (such that $\sum_i \alpha_i^2 = 1$), but which does not affect R. For $\kappa = 1$, eqs. (3) are equivalent to equivalent to eqs. (5) of the main manuscript. To verify that the set of α_i computed via eqs. (3) defines a local maximum in R^2, the eigenvalues of the Hessian $H_{k\ell}$ of R^2 must be non-positive at the maximum. A straightforward calculation yields

$$H_{k\ell}|_{\max} = \left.\frac{\partial^2 [R^2]}{\partial \alpha_k \partial \alpha_\ell}\right|_{\max} = -2\kappa^2 \left[\gamma_{k\ell} - \frac{c_k c_\ell}{\sum_i \kappa \alpha_i c_i} \right], \tag{4}$$

where $|_{\max}$ indicates that the α_i were computed according to eqs. (3). The eigenvalues of $H_{k\ell}|_{\max}$ can be computed numerically which has been implemented into the FMA tool. For the examples presented in this study all eigenvalues of $H_{k\ell}|_{\max}$ were negative, always with the exception of one zero eigenvalue corresponding the the scaling of all α_i by a scalar factor. This finding is expected because R is invariant to a scaling of the coefficients α_i.

Construction of best linear model $m_f(t)$

Let $D = \left\langle [f - m_f]^2 \right\rangle$ denote the MSD between the functional quantity $f(t)$ and its model $m_f(t) = \langle f \rangle + \sum_{i=1}^{d} \beta_i (p_i(t) - \langle p_i \rangle)$. D is minimized with respect to the d parameters β_i by setting the d partial derivatives $\partial D / \partial \beta_\ell$ to zero, immediately yielding eqs. (7) of the main manuscript. It is straightforward to show that the second derivatives of D are twice the elements of the covariance matrix of the principal

components $p_i(t)$, i.e., $\partial^2 D/\partial\beta_\ell\partial\beta_k = 2\text{cov}(p_\ell, p_k)$. Because covariance matrices are positive semidefinite [1], the set of β_i defined by eqs. (7) of the main manuscript correspond to a local minimum of D.

References

1. Mardia KV, Kent JT, Bibby JM (1979) Multivariate Analysis. San Diego: Academic Press.
