# Supplementary material for: Detection of Functional Modes in Protein Dynamics
Source: PLoS Comput Biol. 2009 Aug 28;5(8):e1000480. doi: 10.1371/journal.pcbi.1000480 (PMC2721685; doi:10.1371/journal.pcbi.1000480)
Supplement: Text S2 — FMA of the end-to-end distance of an α-helix (1.92 MB PDF) [file pcbi.1000480.s002.pdf]

Text S2

FMA of the end-to-end distance of an α-helix

As a first and trivial example we analyze collective motions related to the end-to-end distance L_h of the Fs_{21} helix. Because parts of the results are intuitively clear, this example is particularly useful to illustrate the technique. Here, L_h is used as the functional quantity $f(t)$, and it was measured from the distance between the C_α atoms of residues Ala3 and Arg19, as shown in fig. 1A. (Figure numbers in this supporting text S2 always refer to the figures within text S2.) The Pearson coefficient R turned out to be sufficient as correlation measure.

The basis set $\{\mathbf{e}_i\}$ for the collective vector $\mathbf{a}$ was taken from a PCA of the backbone atoms of residues Ala3 to Arg19. The first 20 PCA vectors were used as basis set. The motions along some of the PCA vectors are depicted in fig. 1B. Noteworthy, the PCA vectors correspond to the harmonic modes of a simple helical spring. Vectors 1 and 2 correspond to bending modes (in two dimensions), vector 3 to a torsional and vector 6 to an elongation mode. The other vectors correspond to higher harmonics of these three fundamental modes.

The helix end-to-end distance from the 53-ns trajectory of the completely folded helix is shown in fig. 1C as black curve. The first 40 ns (40.000 frames) were used as model building set (fig. 1C, red background), i.e., to optimize the correlation R_m using eqs. 5 of the main manuscript and to fit the model $m_f(t)$ to the data $f(t)$ using eqs. 7. The left inset in fig. 1C displays in high time resolution the data $f(t)$ and the model $m_f(t)$ as black and red curve, respectively. As apparent from the inset, $m_f(t)$ is a good approximation to $f(t)$. This fact is further quantified in fig. 1D which plots the model $m_f(t)$ versus the data $f(t)$ as scatter plot. A strong correlation of $R_m = 0.98$ between data and model is found.

The remaining 13 ns of the 53-ns trajectory were used for cross-validation (green background in fig. 1C). To this end, the model derived using the model building set was used to predict L_h. The right inset in fig. 1C shows the data and the prediction as black and green curve, respectively. Excellent agreement is found, indicating that the model derived using the model building set has indeed predictive power. The predictive power is quantified in fig. 1E which plots the prediction versus the data. As for the model building set, a strong correlation of $R_c = 0.97$ is found. Noteworthy, data points around $L_h \approx 1.8$ nm are well predicted although such structures were not present in the model building set (fig. 1D).

How many PCs are necessary to construct a good model for L_h? This question is addressed in fig. 1F which plots the correlation R_c of the cross-validation set as a function of the number of PCA vectors d used to construct the model. Apparently, using 6 PCs already yields a reasonable model ($R_c > 0.88$), and using more than 13 PCs yields an excellent model ($R_c > 0.96$). In this trivial example no overfitting occurred, even when using all 153 PCs (data not shown). The choice for d is therefore partly arbitrary in this case. Besides the influence of the number of PCs, the number of simulation frames that are required to construct a good model may be of interest. Figure S2A plots R_c and R_m (using 20 PCA vectors as basis set) as a function of the number of frames in the model building set. As visible from fig. S2A, 30 frames would have been sufficient to construct a good model, i.e. much less than highlighted in fig. 1C.

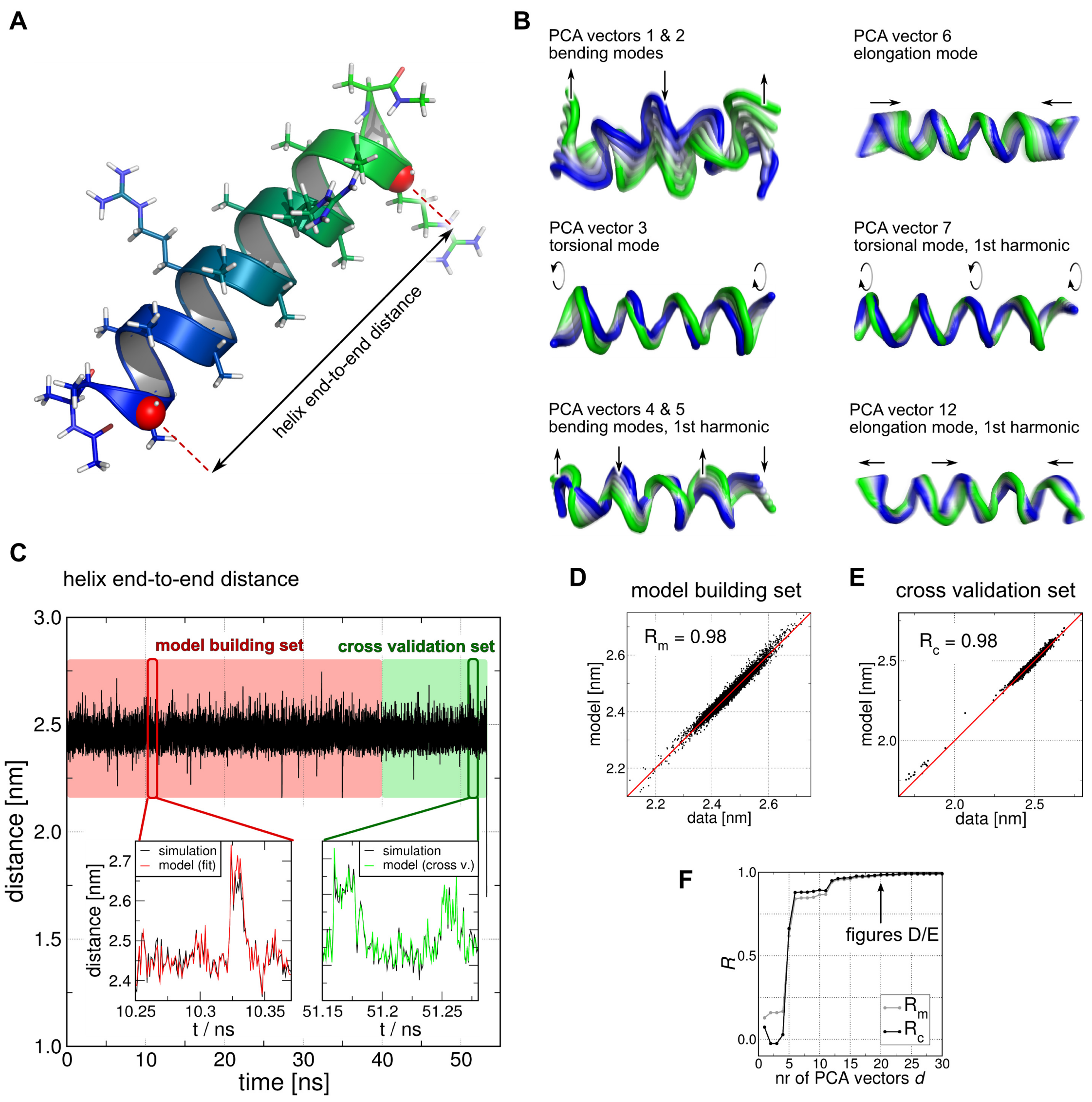

Figure 1. Functional mode analysis of the helix end-to-end distance. (A) The Fs_{21} helix. The end-to-end distance was measured as the distance between the C_α atoms of Ala3 and Arg19 (red spheres). (B) The PCA vectors $\mathbf{e}_i$ of the helix correspond to the harmonic modes of a helical spring, i.e. to bending, torsional, and elongation modes. (C) Helix end-to-end distance as a function of simulation time (black line). The model building set ($t < 40$ ns) is highlighted by a red background, the cross-validation set by a green background. The insets display in fine time scale the simulation data as black, the model as red and green line, respectively. (D) Data versus model for the model building set, and (E) for the cross-validation set. (F) Correlation for cross-validation R_c set as a function of the number of principal components d used in the optimization: $d \geq 6$ yields a good model, $d \geq 12$ an excellent model.

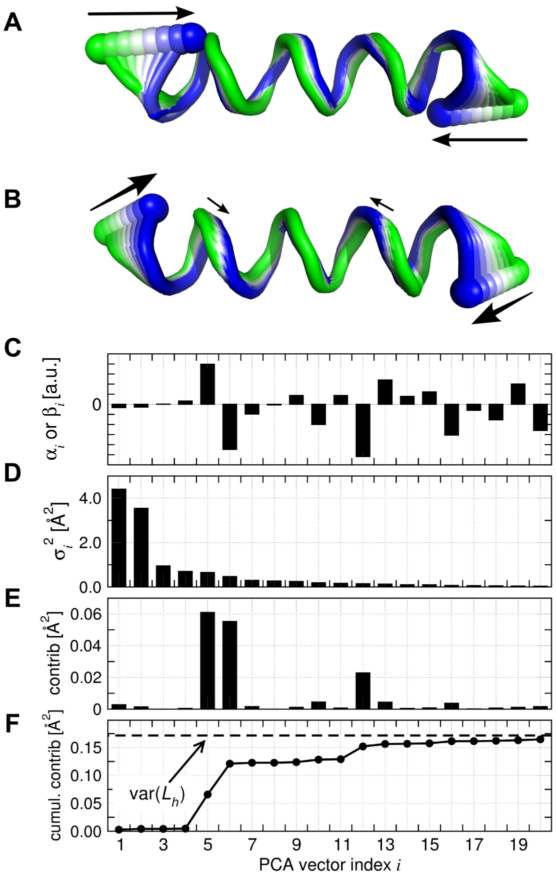

Figure 2. Collective motion related to the helix end-to-end distance L_h. (A) Cartoon representation of the maximally correlated motion (MCM) along the collective vector $\mathbf{a}$, and (B) of the ensemble-weighted MCM contributing to L_h. (C) Components α_i of $\mathbf{a}$ with respect to the PCA vectors $\mathbf{e}_i$, or, alternatively, components β_i of the linear model for L_h. (D) Variances σ_i^2 of the principal components, (E) contribution of principal component i to the variance of the model, and (F) the cumulative contribution of principal component i to the variance of the model. The dashed line indicates the variance var(L_h) of the simulated helix end-to-end distance.

The 'maximally correlated motion' (MCM) along $\mathbf{a}$ and the ensemble-weighted MCM (ewMCM) are visualized in figs. 2A and B, respectively. The vector $\mathbf{a}$ describes the motion of the C_α atoms of Ala3 and Arg19 (shown as spheres) along the helix axis. This result is expected because the helix end-to-end distance was originally measured as the distance between these two atoms. In contrast, the ewMCM is characterized by a combination of collective bending and stretching motions, and is in accordance with the topology of the helix. In that example, the scalar product between the MCM and the normalized ewMCM equals 0.71, indicating that the MCM and the ewMCM are related but not identical. The coordinates α_i of $\mathbf{a}$ with respect to the PCA vectors $\mathbf{e}_i$ are shown in fig. 2C. (Note that $\mathbf{a} = \sum_{i=1}^{d} \alpha_i \mathbf{e}_i$.)

Which PCs contribute to L_h? This question is analyzed in fig. 2D-F. For comparison, fig. 2D displays the variances of the PCs var(p_i), i.e. the contribution of the ith PC to the MSF of the atom positions. The contribution of the PCs to the variance of L_h is plotted in fig. 2E, as derived by eq. 8. The components 4 and 5 (1st harmonic of the bending mode, compare fig. 1B) as well as components 6 and 12 (elongation mode and its first harmonic) dominate the variance of $f(t)$. Noteworthy, the first three PCs (fundamental bending modes and torsional mode) hardly contribute to L_h, although they dominate the MSF of the atom positions. Finally, fig. 2F displays the cumulative contribution of the PCs to var(L_h). Here, 13 components are sufficient to explain the variance of L_h.
