# Supplementary figures and images for: Detection of Functional Modes in Protein Dynamics
Source: PLoS Comput Biol. 2009 Aug 28;5(8):e1000480. doi: 10.1371/journal.pcbi.1000480 (PMC2721685; doi:10.1371/journal.pcbi.1000480)

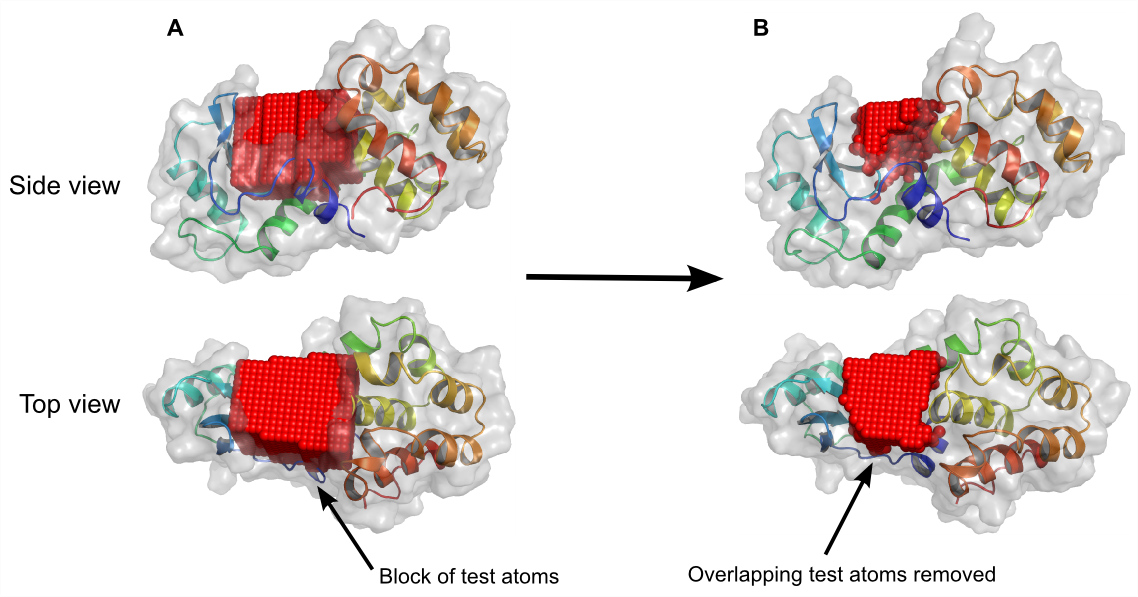

Supplement: Figure S1 — Estimation of the volume of the lysozyme catalytic cleft. A block of test atoms with the approximate shape of the binding site was set up by placing the test atoms on a grid of spacing 1 Å (red block in fig. S1A). The block was placed into the catalytic cleft of a reference structure of T4 lysozyme (T4L). Each T4L structure from the simulation trajectory was fitted onto the reference structure using a least square fit on the backbone atoms. Figure S1A shows one example of a fitted structure, together with the block of test atoms. Subsequently, the test atoms which overlapped with the fitted structure were removed with the genbox tool (fig. S1B). Every remaining atom contributed 1 Å3 to the cleft volume. (0.27 MB JPG) [file pcbi.1000480.s003.jpg]

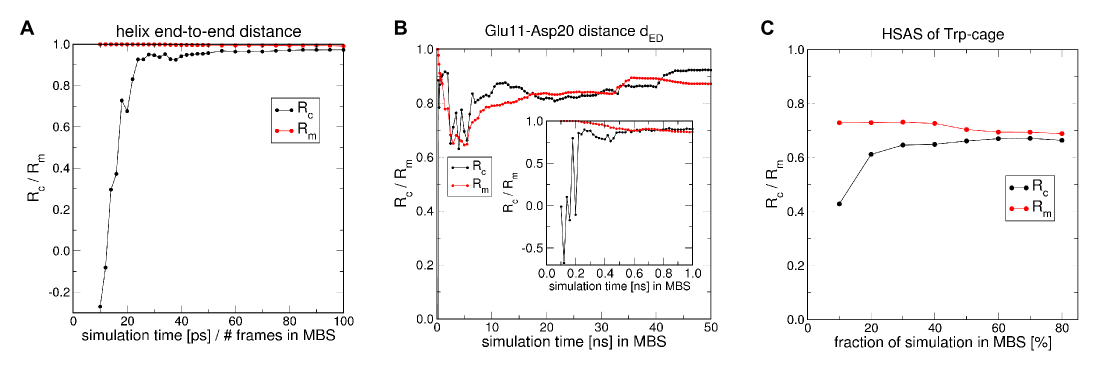

Supplement: Figure S2 — Convergence of FMA with simulation time. (A–C) Correlations R c (black curves) and R m (red curves) of the cross validation and model building sets, respectively, as a function of the number of frames (or simulation time) in the model building set (MBS). (A) Helix end-to-end distance L h. Approximately 30 frames are sufficient to construct a reliable model for L h, as visible from the R c curve (compare Text S2). (B) T4 lysozyme Glu11-Asp20 distance d ED. Approximately 10 ns of simulation are sufficient to yield reasonable models for V cleft and d ED, although the model quality as measured by R c may slightly increase when applying more than 40 ns as MBS. (C) Hydrophobic solvent-accessible surface (HSAS) of Trp-cage in the folded state. From the folded states of the 8 Trp-cage simulations (yellow background in Fig. 5B), an increasing fraction (e.g. 20%) was used as model building set, whereas the remaining fraction (e.g. 80%) of the folded states were applied as cross validation set. As visible from the black R c curve, applying more than 30% of the simulation hardly improves the prediction for the remaining frames. The respective plots for the lysozyme cleft volume and the RMSD of leucine-binding protein are shown in Figs. 3 and 8F, respectively. (0.06 MB PNG) [file pcbi.1000480.s004.png]

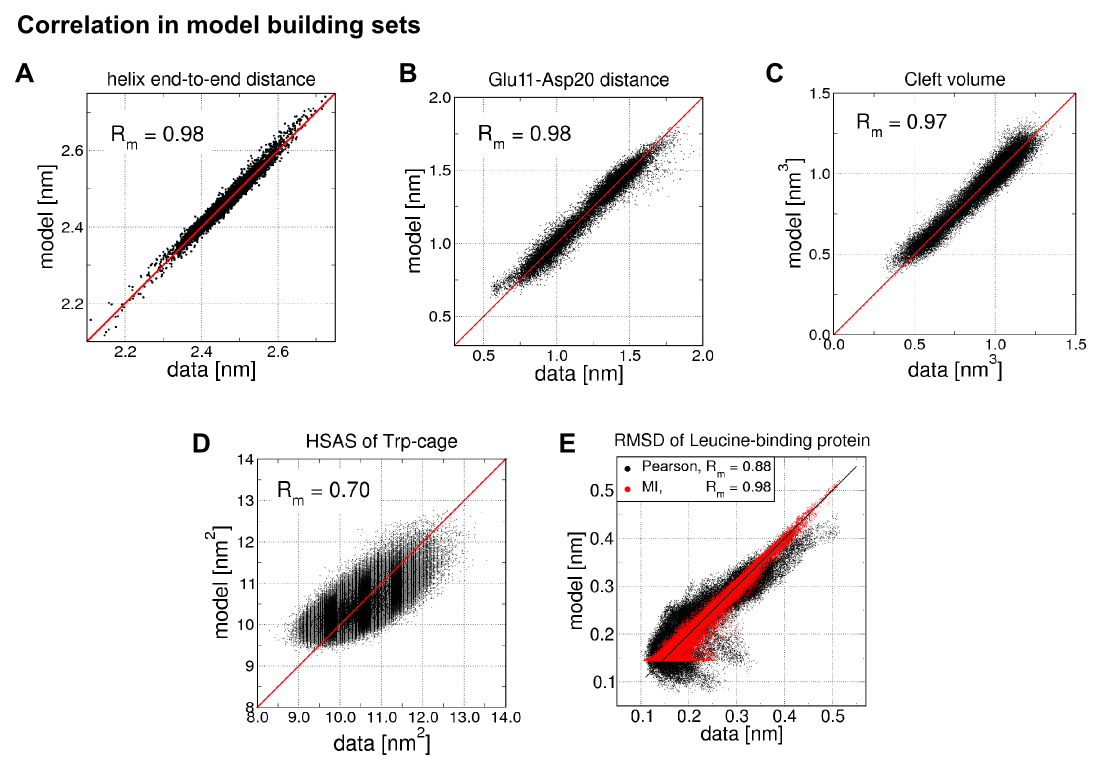

Supplement: Figure S3 — Scatter plots showing model versus data of the model building sets. (A) Helix end-to-end distance, (B) Glu11-Asp20 distance of T4 lysozyme (T4L), (C) cleft volume of T4L, (D) hydrophobic solvent-accessible surface of Trp-cage, and (E) RMSD of backbone atoms of leucine-binding protein (LBP) with respect to its apo structure. Optimization of the mutual information (MI, red dots) yields larger correlation than optimization of Pearson's coefficient (black dots). (0.26 MB PNG) [file pcbi.1000480.s005.png]

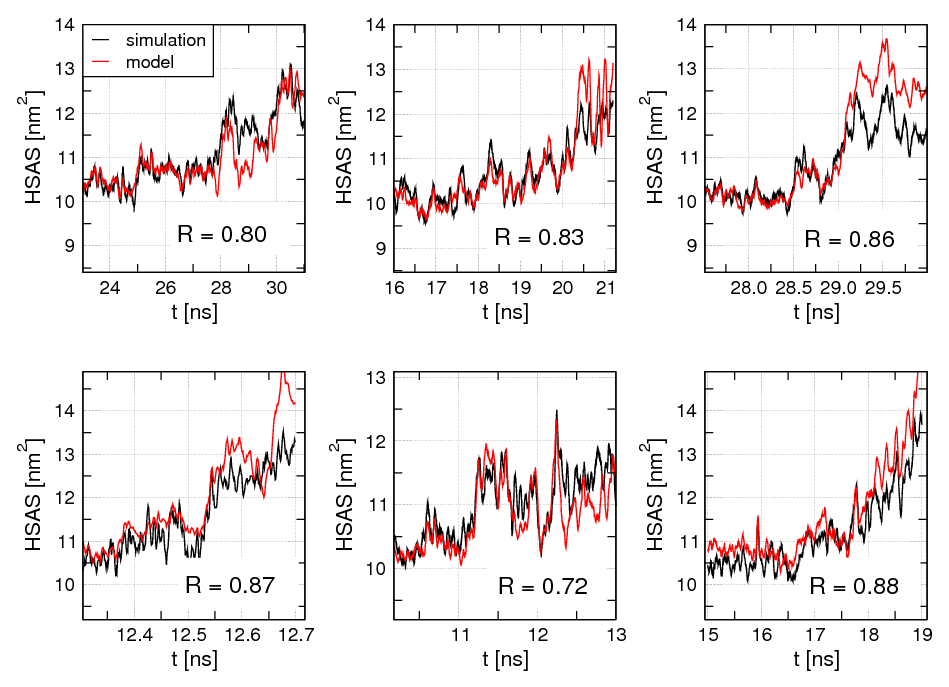

Supplement: Figure S4 — Predictive power of the model for the hydrophobic solvent-accessible surface (HSAS) during six initial unfolding events. HSAS (black curves) during unfolding events and the prediction for the HSAS by the model (red curves). Note that the model was derived only from fluctuations in the folded state. The correlation R between model and data (printed as insets) lies in the range of 0.72 to 0.88. To facilitate the comparison between data and model in these plots, all HSAS curves were slightly smoothed by running averages. The R-values were computed from the non-smoothed data (not shown). (0.12 MB PNG) [file pcbi.1000480.s006.png]
